# Early Warning Scores With and Without Artificial Intelligence

**DOI:** 10.1001/jamanetworkopen.2024.38986

**Published:** 2024-10-15

**Authors:** Dana P. Edelson, Matthew M. Churpek, Kyle A. Carey, Zhenqiu Lin, Chenxi Huang, Jonathan M. Siner, Jennifer Johnson, Harlan M. Krumholz, Deborah J. Rhodes

**Affiliations:** 1 Section of Hospital Medicine, University of Chicago, Chicago, Illinois; 2 AgileMD, San Francisco, California; 3 Section of Pulmonary and Critical Care Medicine, University of Wisconsin School of Medicine and Public Health, Madison; 4 Section of Cardiovascular Medicine, Yale School of Medicine, Yale University, New Haven, Connecticut; 5 Section of Pulmonary, Critical Care and Sleep Medicine, Yale School of Medicine, New Haven, Connecticut; 6 Care Signature, Yale New Haven Health, New Haven, Connecticut; 7 Section of General Internal Medicine, Yale School of Medicine, New Haven, Connecticut

## Abstract

**Importance:**

Early warning decision support tools to identify clinical deterioration in the hospital are widely used, but there is little information on their comparative performance.

**Objective:**

To compare 3 proprietary artificial intelligence (AI) early warning scores and 3 publicly available simple aggregated weighted scores.

**Design, Setting, and Participants:**

This retrospective cohort study was performed at 7 hospitals in the Yale New Haven Health System. All consecutive adult medical-surgical ward hospital encounters between March 9, 2019, and November 9, 2023, were included.

**Exposures:**

Simultaneous Epic Deterioration Index (EDI), Rothman Index (RI), eCARTv5 (eCART), Modified Early Warning Score (MEWS), National Early Warning Score (NEWS), and NEWS2 scores.

**Main Outcomes and Measures:**

Clinical deterioration, defined as a transfer from ward to intensive care unit or death within 24 hours of an observation.

**Results:**

Of the 362 926 patient encounters (median patient age, 64 [IQR, 47-77] years; 200 642 [55.3%] female), 16 693 (4.6%) experienced a clinical deterioration event. eCART had the highest area under the receiver operating characteristic curve at 0.895 (95% CI, 0.891-0.900), followed by NEWS2 at 0.831 (95% CI, 0.826-0.836), NEWS at 0.829 (95% CI, 0.824-0.835), RI at 0.828 (95% CI, 0.823-0.834), EDI at 0.808 (95% CI, 0.802-0.812), and MEWS at 0.757 (95% CI, 0.750-0.764). After matching scores at the moderate-risk sensitivity level for a NEWS score of 5, overall positive predictive values (PPVs) ranged from a low of 6.3% (95% CI, 6.1%-6.4%) for an EDI score of 41 to a high of 17.3% (95% CI, 16.9%-17.8%) for an eCART score of 94. Matching scores at the high-risk specificity of a NEWS score of 7 yielded overall PPVs ranging from a low of 14.5% (95% CI, 14.0%-15.2%) for an EDI score of 54 to a high of 23.3% (95% CI, 22.7%-24.2%) for an eCART score of 97. The moderate-risk thresholds provided a median of at least 20 hours of lead time for all the scores. Median lead time at the high-risk threshold was 11 (IQR, 0-69) hours for eCART, 8 (IQR, 0-63) hours for NEWS, 6 (IQR, 0-62) hours for NEWS2, 5 (IQR, 0-56) hours for MEWS, 1 (IQR, 0-39) hour for EDI, and 0 (IQR, 0-42) hours for RI.

**Conclusions and Relevance:**

In this cohort study of inpatient encounters, eCART outperformed the other AI and non-AI scores, identifying more deteriorating patients with fewer false alarms and sufficient time to intervene. NEWS, a non-AI, publicly available early warning score, significantly outperformed EDI. Given the wide variation in accuracy, additional transparency and oversight of early warning tools may be warranted.

## Key Points

**Question:**

How do hospital early warning scores compare with one another?

**Findings:**

In this cohort study that compared 6 early warning scores across 362 926 patient encounters, eCARTv5, a machine learning model, identified clinical deterioration best with an area under the receiver operating characteristics curve (AUROC) of 0.895 and the highest positive predictive values at both the moderate- and high-risk matched thresholds. The National Early Warning Score, a non–artificial intelligence score with an AUROC of 0.831, was the second-best performer at both thresholds, while the Epic Deterioration Index was one of the worst, with an AUROC of 0.808 and the lowest positive predictive values.

**Meaning:**

Given the wide variation in accuracy, these findings suggest that additional transparency and oversight of early warning tools may be warranted.

## Teaser

This cohort study compares 3 proprietary artificial intelligence early warning score and 3 publicly available simple aggregated weighted scores for identifying clinical deterioration in hospitalized patients.

## Introduction

Clinical deterioration occurs in up to 5% of hospitalized patients, and delays in escalation of care for these patients are common and associated with increased mortality and length of stay.^1,2,3,4^ Early warning scores, designed to help clinicians recognize deterioration earlier, have grown in numbers and complexity with the advent of electronic health records (EHRs) and the rapid evolution of artificial intelligence (AI).^5^ These scores can be deployed inside EHRs, and a few have been associated with decreased mortality.^6,7^ Hospitals now have several options for early warning scores, ranging in complexity from transparent aggregated weighted scores, like the Modified Early Warning Score (MEWS) and the National Early Warning Score (NEWS), to AI-based models increasing in complexity and opacity from logistic regression to advanced statistical models such as gradient-boosted machine models and neural networks, to name a few.^8,9,10,11,12,13,14,15,16^

The US Food and Drug Administration (FDA) has indicated that AI early warning scores constitute medical devices subject to federal oversight, and President Biden recently issued an executive order on AI, which included direction to “advance the responsible use of AI in health care.”^17,18,19^ However, only 2 general early warning scores have been cleared by the FDA to date, namely the Rothman Index (RI)^20^ and eCARTv5 (eCART).^21^ Meanwhile, the Epic Deterioration Index (EDI), the most widely available of such scores, has not been formally vetted.^22^

Further, despite their widespread use, the relative performance of many of these scores is unknown due to the paucity of direct comparisons and a lack of defined performance targets, leaving health systems to select models without comparative data.^5,23,24^ This issue is critical because tools that fail to recognize deterioration may provide a false sense of security to staff, and those with high false alarm rates are likely to be ignored or, worse, could divert scarce resources like nursing attention and critical care beds from other patients who need them more.^25^ Therefore, we sought to perform a head-to-head comparison of 3 proprietary AI early warning scores (RI,^26^ eCART,^27^ and EDI^28^) and 3 publicly available simple aggregated weighted scores (MEWS, NEWS, and NEWS2) for identifying clinical deterioration.

## Methods

### Overview

We conducted a retrospective cohort study comparing the 6 scores in adult patients on medical-surgical wards admitted to 7 hospital campuses in the Yale New Haven Health System, which included 2 major academic, 3 community teaching, and 2 nonteaching community medical centers. eTables 1 and 2 in Supplement 1 describe the study models. Five of the models were selected a priori and NEWS2 was added subsequently. All tested models were included in the reporting. The study was approved by the University of Chicago and Yale New Haven Health System institutional review boards with a waiver of informed consent on the basis of minimal risk and general impracticability, and the report follows the Strengthening the Reporting of Observational Studies in Epidemiology (STROBE) reporting guideline for cohort studies.

### Population

A convenience sample of consecutive medical-surgical hospitalizations occurring in patients 18 years or older at 1 of 7 hospital campuses between March 9, 2019, and November 9, 2023, was included. Patients discharged from the emergency department without being admitted to the hospital were excluded, as were patients whose hospitalizations never included a medical-surgical ward stay. Patients receiving palliative care were not specifically excluded. Demographic characteristics such as age, race, sex, and Elixhauser comorbidities defined by billing codes^29^ were included to demonstrate the diversity among the hospital campuses. Rapid response team (RRT) nurses received mobile alerts at 4 of the campuses (A, D, E, and G) (see eTable 1 in Supplement 1 for workflow specifics).

### Outcomes

The primary outcome was clinical deterioration, defined as death on a medical-surgical ward or direct transfer from a ward to an intensive care unit (ICU) occurring within 24 hours of a score, a commonly used end point in validation studies of early warning scores.^5^ Secondary outcomes included deterioration within 12 and 48 hours and death within 12, 24, and 48 hours of a score. Death was determined using the discharge disposition from the admission, discharge, and transfer data feed in the clinical record, with the time of death being the last recorded vital sign, independently of whether an ICU transfer preceded it. ICU transfer was determined using the transfer disposition from the admission, discharge, and transfer data feed in the clinical record, with the time of transfer being the last recorded vital sign in a medical-surgical location.

### Statistical Analysis

All RI and EDI scores documented in medical-surgical wards were included. eCART, MEWS, NEWS, and NEWS2 scores were retrospectively calculated any time a new eCART variable resulted in the EHR. The EDI score produced more frequent observations than the rest of the scores. Therefore, to enable head-to-head comparison of the scores, we matched observation times for each score where observations for MEWS, NEWS, NEWS2, eCART, and RI were carried forward to align with each documented EDI score. A 100-iteration bootstrapped analysis randomly selected 1 observation per encounter, and the performance of scores was assessed by the area under the receiver operating characteristic curve (AUROC) with 95% CIs and compared using the DeLong test.^30^ Threshold scores with a sensitivity closest to that of a NEWS score of 5 at the observation level were selected as the moderate-risk trigger, while threshold scores with a specificity closest to that of a NEWS score of 7 were selected as the high-risk trigger for all scores. We selected NEWS as the comparator rather than NEWS2 because the latter has not been shown to improve discrimination over NEWS.^31^ In calculating NEWS2, we used the Spo_2_ scale 1 for all observations in an encounter preceding a Paco_2_ value of greater than 45 mm Hg with a simultaneous Fio_2_ value of greater than 21%. Thereafter we used the Spo_2_ scale 2 through discharge. Sensitivity, specificity, positive predictive value (PPV), and negative predictive value were calculated for each threshold of each score. Precision recall curves were constructed to show the association between PPV and sensitivity at the observation level. Efficiency curves were constructed to show the association between frequency of elevation and sensitivity at the encounter (ie, admission) level, using the highest score occurring before the first deterioration event or before discharge for those who never had a deterioration event during their hospitalization. Descriptive statistics were used to describe patient demographic characteristics. Finally, we calculated the median trigger to event time at the moderate- and high-risk threshold of each model and compared them using a Wilcoxon rank sum, imputing a median time of 0 hours for any event that never met the threshold. Analyses were performed using Stata, version 16.1 (StataCorp LLC), and R, version 4.2.1 (R Project for Statistical Computing). Two-tailed *P* < .05 was considered statistically significant.

## Results

The study population included 362 926 inpatient encounters with a median patient age of 64 (IQR, 47-77) years; 200 642 patients (55.3%) were female and 162 284 (44.7%) were male. In terms of race, 1184 patients (0.3%) were American Indian or Alaska Native; 6878 (1.9%), Asian; 61 093 (16.8%), Black; 783 (0.2%), Native Hawaiian or other Pacific Islander; 250 866 (69.1%), White; and 42 122 (11.6%), declined or unknown. Of these, 16 693 patients (4.6%) were transferred to an ICU from a ward or died on a ward. The median hospital length of stay was 96 (IQR, 59-171) hours. Additional demographic characteristics are shown in Table 1 and demonstrate considerable variation across the 7 hospital campuses, with campus F having the highest median age at 73 (IQR, 61-83) years, the highest percentage of White patients (95.0%), and the highest rates of ICU transfer (5.5%) and mortality (3.0%) and Campus G having the lowest median age at 61 (IQR, 42-74) years and the highest rates of cancer (21.0%) and surgery (45.6%).

**Table 1.** Demographic Characteristics

Characteristic	Patient encounters by campus, No. (%)							
	All (N = 362 926)	A (n = 63 783)	B (n = 32 352)	C (n = 40 562)	D (n = 10 130)	E (n = 62 457)	F (n = 10 944)	G (n = 142 698)
Age, median (IQR), y	64 (47-77)	64 (46-78)	65 (39-81)	66 (47-78)	72 (59-82)	66 (54-78)	73 (61-83)	61 (42-74)
Hospital length of stay, median (IQR), h	96 (59-171)	100 (63-176)	78 (52-133)	92 (55-161)	93 (55-152)	105 (63-184)	91 (55-143)	98 (59-186)
Race								
American Indian or Alaska Native	1184 (0.3)	104 (0.2)	33 (0.1)	261 (0.6)	17 (0.2)	276 (0.4)	42 (0.4)	451 (0.3)
Asian	6878 (1.9)	780 (1.2)	1033 (3.2)	685 (1.7)	126 (1.2)	634 (1.0)	78 (0.7)	3542 (2.5)
Black or African American	61 093 (16.8)	14 232 (22.3)	1934 (6.0)	3371 (8.3)	738 (7.3)	16 741 (26.8)	204 (1.9)	23 873 (16.7)
Native Hawaiian or Other Pacific Islander	783 (0.2)	116 (0.2)	58 (0.2)	88 (0.2)	11 (0.1)	167 (0.3)	10 (0.1)	333 (0.2)
White	25 0866 (69.1)	35 452 (55.6)	23 924 (73.9)	32 462 (80.0)	8806 (86.9)	39 287 (62.9)	10 399 (95.0)	100 536 (70.5)
Declined or unknown	42 122 (11.6)	13 099 (20.5)	5370 (16.6)	3695 (9.1)	432 (4.3)	5352 (8.6)	211 (1.9)	13 963 (9.8)
Sex								
Female	200 642 (55.3)	35 162 (55.1)	20 613 (63.7)	23 001 (56.7)	5452 (53.8)	33 666 (53.9)	5591 (51.1)	77 157 (54.1)
Male	162 280 (44.7)	28 620 (44.9)	11 739 (36.3)	17 561 (43.3)	4678 (46.2)	28 790 (46.1)	5353 (48.9)	65 539 (45.9)
Declined or unknown	4 (0.01)	1 (0.002)	0	0	0	1 (0.002)	0	2 (0.001)
Congestive heart failure	83 873 (23.1)	15 655 (24.5)	4625 (14.3)	9382 (23.1)	2554 (25.2)	17 990 (28.8)	3361 (30.7)	30 306 (21.2)
Chronic pulmonary disease	104 305 (28.7)	18 859 (29.6)	6298 (19.5)	13 035 (32.1)	3010 (29.7)	21 457 (34.4)	3850 (35.2)	37 796 (26.5)
Kidney failure	73 575 (20.3)	13 586 (21.3)	4679 (14.5)	8624 (21.3)	2257 (22.3)	15 422 (24.7)	2886 (26.4)	26 121 (18.3)
Liver disease	32 789 (9.0)	5578 (8.7)	1742 (5.4)	3513 (8.7)	782 (7.7)	6109 (9.8)	1610 (14.7)	13 455 (9.4)
Metastatic cancer	18 456 (5.1)	2687 (4.2)	1194 (3.7)	1431 (3.5)	269 (2.7)	1567 (2.5)	521 (4.8)	10 787 (7.6)
Solid tumor without metastasis	33 771 (9.3)	5110 (8.0)	2206 (6.8)	2735 (6.7)	506 (5.0)	3098 (5.0)	1000 (9.1)	19 116 (13.4)
Obesity	92 337 (25.4)	16 928 (26.5)	5882 (18.2)	11 125 (27.4)	2974 (29.4)	19 746 (31.6)	2589 (23.7)	33 093 (23.2)
Surgery during the encounter	144 227 (39.7)	21 820 (34.2)	12 361 (38.2)	14 009 (34.5)	3188 (31.5)	24 883 (39.8)	2902 (26.5)	65 064 (45.6)
Ward to ICU transfer	13 179 (3.6)	2104 (3.3)	436 (1.3)	1826 (4.5)	512 (5.1)	1786 (2.9)	600 (5.5)	5915 (4.1)
Mortality	7600 (2.1)	1751 (2.7)	689 (2.1)	1021 (2.5)	212 (2.1)	1038 (1.7)	331 (3.0)	2558 (1.8)

Abbreviation: ICU, intensive care unit.

AUROCs for identifying clinical deterioration within 24 hours of an observation are shown in Table 2, and the ROC curves are shown in the eFigure in Supplement 1. Across the whole population, eCART had the highest AUROC at 0.895 (95% CI, 0.891-0.900), followed by NEWS2 at 0.831 (95% CI, 0.826-0.836), NEWS at 0.829 (95% CI, 0.824-0.835), RI at 0.828 (95% CI, 0.823-0.834), EDI at 0.808 (95% CI, 0.802-0.812), and MEWS at 0.757 (95% CI, 0.750-0.764). This pattern generally held across the 7 hospitals, with eCART consistently outperforming the other scores. NEWS and NEWS2 were statistically indistinct from one another across all the hospitals by AUROC. Secondary outcomes are shown in eTable 3 in Supplement 1. Across the scores, AUROCs were generally highest for mortality at 12 hours and lowest for deterioration at 48 hours, but eCART consistently outperformed the other scores for each outcome. RI was the second-best performer by AUROC for mortality and NEWS2 outperformed NEWS for mortality at 24 and 48 hours, but not at 12 hours.

**Table 2.** AUROC for Identifying Intensive Care Unit Transfer or Death Within 24 Hours by Hospital Campus

Hospital campus	Encounters, No.	AUROC (95% CI)					
		MEWS	EDI	RI	NEWS	NEWS2	eCART
All	362 926	0.757 (0.750-0.764)	0.808 (0.802-0.812)	0.828 (0.823-0.834)	0.829 (0.824-0.835)	0.831 (0.826-0.836)	0.895 (0.891-0.900)
A	63 783	0.788 (0.773-0.800)	0.836 (0.824-0.847)	0.852 (0.842-0.863)	0.848 (0.837-0.861)	0.846 (0.837-0.861)	0.903 (0.895-0.912)
B	32 352	0.806 (0.786-0.827)	0.856 (0.838-0.872)	0.881 (0.869-0.897)	0.870 (0.853-0.888)	0.874 (0.862-0.892)	0.931 (0.919-0.945)
C	40 562	0.740 (0.722-0.754)	0.784 (0.771-0.797)	0.796 (0.783-0.807)	0.796 (0.783-0.807)	0.801 (0.789-0.816)	0.881 (0.871-0.891)
D	10 130	0.720 (0.696-0.752)	0.773 (0.741-0.795)	0.792 (0.770-0.813)	0.798 (0.775-0.822)	0.795 (0.769-0.817)	0.871 (0.850-0.888)
E	62 457	0.747 (0.723-0.765)	0.800 (0.783-0.818)	0.813 (0.795-0.829)	0.831 (0.815-0.851)	0.829 (0.813-0.846)	0.885 (0.872-0.898)
F	10 944	0.736 (0.716-0.752)	0.727 (0.710-0.742)	0.746 (0.729-0.763)	0.784 (0.768-0.801)	0.780 (0.763-0.797)	0.866 (0.853-0.882)
G	142 698	0.744 (0.730-0.755)	0.814 (0.806-0.823)	0.834 (0.828-0.843)	0.828 (0.816-0.838)	0.831 (0.822-0.841)	0.894 (0.887-0.902)

Abbreviations: AUROC, area under the receiver operating characteristics curve; eCART, eCARTv5; EDI, Epic Deterioration Index; MEWS, Modified Early Warning Score; NEWS, National Early Warning Score; RI, Rothman Index.

After matching scores at the moderate-risk sensitivity level for a NEWS score of 5, overall PPVs ranged from a low of 6.3% (IQR, 6.1%-6.4%) for an EDI score of 41 to a high of 17.3% (IQR, 16.9%-17.8%) for an eCART score of 94 (Table 3). Matching scores at the high-risk specificity of a NEWS score of 7 yielded overall PPVs ranging from a low of 14.5% (IQR, 14.0%-15.2%) for an EDI score of 54 to a high of 23.3% (95% CI, 22.7%-24.2%) for an eCART score of 97 (Table 3). The moderate-risk thresholds provided median lead times ranging from 20 (IQR, 0-104) to 31 (IQR, 3-116) hours for all the scores. Median lead time at the high-risk threshold was 11 (IQR, 0-69) hours for eCART, 8 (IQR, 0-63) hours for NEWS, 6 (IQR, 0-62) hours for NEWS2, 5 (IQR, 0-56) hours for MEWS, 1 (IQR, 0-39) hour for EDI, and 0 (IQR, 0-42) hours for RI.

**Table 3.** Observation-Level Test Characteristics at NEWS-Matched Thresholds for Identifying Intensive Care Unit Transfer or Death Within 24 Hours

Risk level	Score	Threshold	Characteristic (95% CI), %				
			Positivity rate	Sensitivity	Specificity	PPV	NPV
Moderate^a^	eCART	94	3.3 (3.3-3.4)	52.2 (51.0-53.4)	97.2 (97.2-97.3)	17.3 (16.9-17.8)	99.5 (99.4-99.5)
	NEWS	5	6.0 (5.9-6.0)	51.5 (50.4-52.8)	94.5 (94.5-94.6)	9.5 (9.3-9.8)	99.4 (99.4-99.5)
	NEWS2	6	6.3 (6.3-6.4)	50.1 (49.0-51.4)	94.2 (94.1-94.2)	8.7 (8.5-9.0)	99.4 (99.4-99.4)
	MEWS	3	5.9 (5.8-5.9)	43.7 (42.5-44.9)	94.6 (94.5-94.6)	8.2 (8.0-8.5)	99.3 (99.3-99.4)
	RI	41	8.2 (8.2-8.3)	51.2 (50.2-52.2)	92.3 (92.2-92.3)	6.9 (6.7-7.0)	99.4 (99.4-99.4)
	EDI	41	8.9 (8.9-9.0)	50.7 (49.4-51.9)	91.5 (91.5-91.6)	6.3 (6.1-6.4)	99.4 (99.4-99.4)
High^b^	eCART	97	2.0 (2.0-2.0)	42.5 (41.4-43.9)	98.4 (98.4-98.5)	23.3 (22.7-24.2)	99.4 (99.3-99.4)
	NEWS	7	1.8 (1.8-1.8)	31.5 (30.7-32.9)	98.5 (98.5-98.5)	19.1 (18.5-20.0)	99.2 (99.2-99.3)
	NEWS2	8	2.1 (2.1-2.2)	30.5 (29.6-32.1)	98.2 (98.1-98.2)	15.8 (15.1-16.4)	99.2 (99.2-99.2)
	MEWS	4	1.8 (1.8-1.8)	27.6 (26.4-28.6)	98.5 (98.5-98.5)	16.9 (16.2-17.5)	99.2 (99.2-99.2)
	RI	24	1.9 (1.8-1.9)	27.9 (27.0-29.0)	98.4 (98.4-98.5)	16.6 (15.9-17.1)	99.2 (99.2-99.2)
	EDI	54	1.8 (1.8-1.9)	24.2 (23.4-25.2)	98.4 (98.4-98.4)	14.5 (14.0-15.2)	99.1 (99.1-99.2)

Abbreviations: eCART, eCARTv5; EDI, Epic Deterioration Index; MEWS, Modified Early Warning Score; NEWS, National Early Warning Score; NPV, negative predictive value; PPV, positive predictive value; RI, Rothman Index.

^a^ Matched to the sensitivity of a NEWS score of 5 for each score at the observation level.

^b^ Matched to the specificity of a NEWS score of 7 for each score at the observation level.

Precision-recall curves and efficiency curves for each model are shown in the Figure, A and B, respectively. Additional observation-level thresholds ranging from a sensitivity of 70% to a specificity of 99% for each score are shown in eTable 4 in Supplement 1 and demonstrate that eCART had consistently higher PPVs for any given sensitivity, while EDI consistently had the lowest. Additional encounter-level thresholds for each score are shown in eTable 5 in Supplement 1. eCART had the highest efficiency as seen by the consistently lower trigger frequency for any given sensitivity. Consistent with the observation-level results, NEWS was the second-best performer of the 6 tools at the encounter level and EDI was among the lowest. At the matched moderate-risk threshold, eCART would have identified 66 fewer deteriorations than NEWS and 304 more than EDI, while alerting on 41 540 and 47 710 fewer patients, respectively (Table 4). At the high-risk threshold, eCART would have identified 1473 more deteriorations than NEWS and 3744 more than EDI, while alerting on 4809 fewer patients than NEWS but 8686 more patients than EDI. However, the median lead time between the first trigger and the first deterioration event at that threshold was 1 (IQR, 0-39) hour for EDI compared with 8 (IQR, 0-63) hours for NEWS and 11 (IQR, 0-69) hours for eCART (*P* < .001), meaning that in over half the deteriorations, an eCART score of 97 would have provided at least 11 hours to intervene, while an EDI score of 58 would have offered only an hour or less. At the moderate-risk threshold, median lead time was highest for NEWS (31 [IQR, 4-110] hours) and EDI (31 [IRQ, 3-116] hours) and lowest for RI (20 [IQR, 0-104] hours; *P* < .001).

**Figure.**
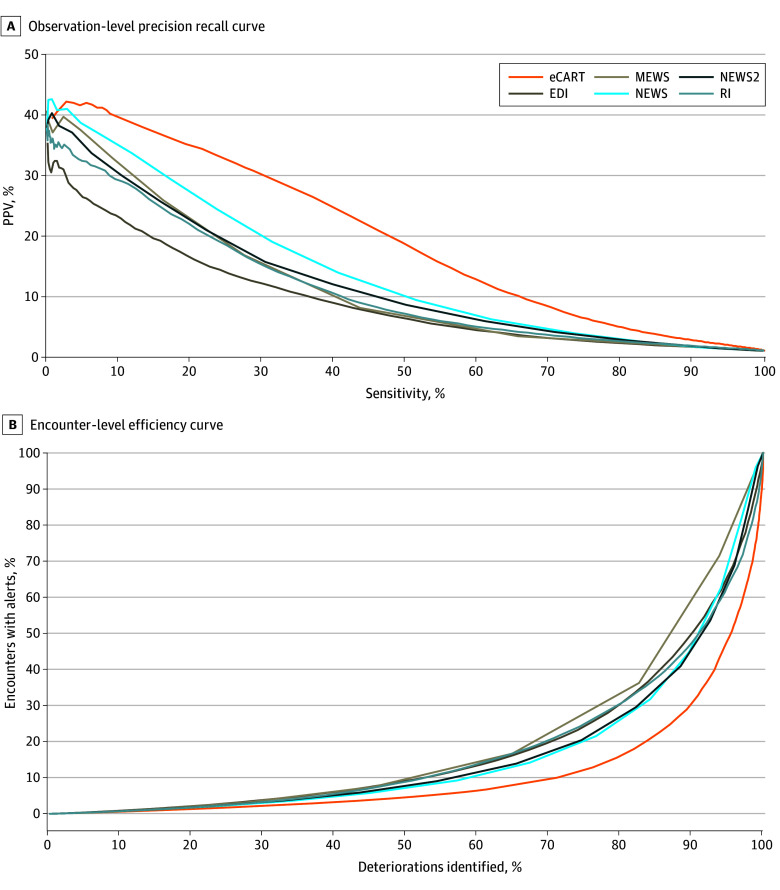
Observation-Level Precision Recall Curve and Encounter-Level Efficiency Curve for Each Score Precision is measured as positive predictive value (PPV); recall, as sensitivity. eCART indicates eCARTv5; EDI, Epic Deterioration Index; MEWS, Modified Early Warning Score; NEWS, National Early Warning Score; and RI, Rothman Index.

**Table 4.** Encounter-Level Comparison With NEWS

Risk	Score	Threshold	Encounters with alerts, No. (%) (N = 362 926)	Trigger differential, No.	Encounters with a deterioration event, No. (%) (n = 16 693)	Catch differential	Lead time, h, median (IQR)	Lead-time *P* value
Moderate^a^	eCART	94	73 761 (20.3)	−41 540	13 989 (83.8)	−66	22 (2-94)	<.001
	NEWS	5	115 301 (31.8)	[Reference]	14 055 (84.2)	[Reference]	31 (4-110)	NA
	NEWS2	6	107 208 (29.5)	−8093	13 719 (82.2)	−336	28 (3-108)	<.001
	MEWS	3	131 572 (36.3)	16 271	13 792 (82.6)	−263	26 (2-106)	<.001
	RI	41	76 287 (21.0)	−39 014	11 805 (70.7)	−2250	20 (0-104)	<.001
	EDI	41	121 471 (33.5)	6170	13 685 (82.0)	−370	31 (3-116)	.40
High^b^	eCART	97	46 682 (12.9)	−4809	12 711 (76.1)	1473	11 (0-69)	<.001
	NEWS	7	51 491 (14.2)	[Reference]	11 238 (67.3)	[Reference]	8 (0-63)	NA
	NEWS2	8	50 512 (13.9)	−979	10 924 (65.4)	−314	6 (0-62)	.009
	MEWS	4	60 547 (16.7)	9056	10 828 (64.9)	−410	5 (0-56)	<.001
	RI	24	29 036 (8.0)	−22 455	7927 (47.5)	−3311	0 (0-42)	<.001
	EDI	54	37 996 (10.5)	−13 495	8967 (53.7)	−2271	1 (0-39)	<.001

Abbreviations: eCART, eCARTv5; EDI, Epic Deterioration Index; MEWS, Modified Early Warning Score; NA, not applicable; NEWS, National Early Warning Score; RI, Rothman Index.

^a^ Matched to the sensitivity of a NEWS score of 5 for each score at the observation level.

^b^ Matched to the specificity of a NEWS score of 7 for each score at the observation level.

## Discussion

In a multicenter cohort study of 362 926 patient encounters, we found that eCART, a gradient-boosted machine learning model, meaningfully outperformed 2 other AI early warning scores (EDI and RI) and 3 aggregated weighted scores (MEWS, NEWS, and NEWS2) in identifying clinical deterioration in the hospital. The more surprising finding was that NEWS, one of the simple tools that can be calculated without a computer, outperformed both the EDI and RI.

The performance differences in these scores were sizeable and could affect patient outcomes and resource allocation. Specifically, compared with EDI, the PPVs of NEWS were at least 20% higher, and for eCART, were more than 60% higher at the moderate- and high-risk thresholds, and there was no threshold where the tradeoff between alerts and deterioration detection was favorable for EDI. Furthermore, the high-risk threshold for EDI had a median lead time of 1 hour compared with 11 hours for eCART and 8 hours for NEWS. While the ideal lead time for a deterioration warning is unknown and likely depends on the level of escalation required, prior data suggest that transfer to an ICU within 4 to 6 hours of meeting deterioration criteria may improve outcomes.^1,2,3,4^

The use of AI-informed clinical decision support has been growing rapidly, with early data suggesting that it can improve patient outcomes and efficiency. Multicenter studies of the Advance Alert Monitor program from Kaiser Permanente and an earlier version of eCART demonstrated an association with decreased mortality in patients at elevated risk following implementation.^6,7^ While the Kaiser mortality benefit was not accompanied by demonstrated process improvement, Winslow et al^6^ showed increased and earlier ICU transfers and more frequent vital sign reassessments following the intervention. These findings suggest that clinicians can modify their behavior to improve patient care in response to a clinical decision support tool, which is consistent with one of the findings in a recent randomized vignette study.^32^ However, Jabbour et al^32^ also showed that clinicians are likely to follow the AI even when it is biased, putting patients at risk.

The FDA recently clarified that software that uses EHR data “to identify signs of patient deterioration and alert an HCP [health care professional]” is a medical device subject to regulation by the FDA.^18^ However, despite their proliferation, very few such devices have been cleared by the FDA.^33,34^ Lee et al^33^ reviewed 521 FDA authorizations for AI and machine learning devices and found only 10 critical care ones, of which approximately half were early warning indices, including RI. That study^33^ and an accompanying editorial^19^ criticized the FDA for lacking peer-reviewed model assessment and reliance on non-AI and non–machine learning predicate devices to establish substantial equivalence. Our study’s wide variation in performance supports the importance of oversight and transparency concerning early warning tools.

Unlike the other models, the development and validation study for EDI has not been peer-reviewed or published,^22^ and external evaluations have been variable in methodology and outcome,^8,9,16^ despite Epic being the fastest-growing EHR vendor in acute care hospitals in the US, with a market share covering 48% of beds in 2022.^35^ Further, Epic incentivizes hospitals to use these algorithms.^36^ The performance of EDI in this study was higher than that demonstrated in 3 other academic studies performed at the University of Minnesota,^9^ the University of Michigan,^8^ and Vanderbilt University^16^ and in two of them,^8,16^ EDI appeared to outperform NEWS. The 3 studies defined deterioration differently, with inclusion of varying outcomes and time frames. For example, Steitz and colleagues^16^ included RRTs in the definition of deterioration, and it is possible that choice would have favored EDI over NEWS and MEWS if clinicians were making decisions to call an RRT with knowledge of the EDI score. Further, all 3 studies included all observations rather than using a bootstrapped approach, which resulted in lower AUROCs across the board compared with the bootstrapped results in our analysis. Despite EDI’s retrospective performance, a small clinical implementation study at Stanford University^37^ found that patients crossing the alert threshold of 65 appeared less likely to require care escalations, defined as RRT activations, ICU transfer, or cardiac arrest compared with those who did not cross the threshold. However, it is worth noting that in our study, an EDI threshold of 65 would only have picked up 30% of the deterioration events.

There are 2 likely reasons for the higher performance of eCART compared with the other models. First, eCART relies on a gradient-boosting machine framework, which handles interaction and missing variables and has been shown to outperform many other AI models for predicting clinical deterioration.^38^ Second, it includes dozens of additional inputs, particularly trend variables, which have also been shown to improve model performance and likely decrease false alarms associated with chronic but stable physiological abnormalities common in hospitalized patients, such as atrial fibrillation and end-stage kidney disease.^38,39^ In fact, 2 of the most heavily weighted variables in eCART, namely the maximum respiratory rate and minimum systolic blood pressure in the prior 24 hours, are not included in any of the other models.^27^ In addition, supplemental oxygen requirement, the most heavily weighted variable in eCART, is missing from both MEWS and RI.

One of the complicating issues of comparing models, and a byproduct of limited FDA guidance, is the selection of comparable thresholds and target performance metrics. This report supports the use of NEWS as a standard comparator because it is publicly available, performs well, and has clearly defined moderate- and high-risk thresholds that can be used to match on sensitivity and specificity, respectively. Byrd and colleagues^9^ argued for using a sensitivity of at least 50% for the moderate-risk threshold and a PPV of at least 10% for the high-risk threshold, which all of the scores in this study met, except a MEWS score of 3. However, we suggest that a more reliable approach is to match the higher-granularity tool to NEWS, using the specificity at the upper threshold and the sensitivity at the lower threshold and comparing PPV, to avoid the risk of having NEWS fail to match an arbitrary threshold. Regardless, the provision of full test characteristics tables at all thresholds is critical to full transparency, neither of which, to our knowledge, has been shared publicly for either RI or EDI prior to this study.

### Strengths and Limitations

Our study has several strengths. First it is, to our knowledge, the largest published study of both EDI and RI. Second, it was conducted in 7 demographically distinct and variably resourced hospital campuses across 4 and one-half years, both before and after the COVID-19 pandemic, increasing the likelihood of the results being generalizable. Additionally, it used a bootstrapped analysis that avoided the inclusion of repeat observations in the same encounter.

The study also has several limitations. First, eCART, MEWS, and NEWS were retrospectively calculated, while EDI and RI were prospectively calculated. Execution of scoring in a clinical setting might impact the calculations, or the display of RI and EDI scores to clinicians might impact the measured outcomes, further complicating the generalizability of comparative analyses. However, the consistency of the results between the campuses that deployed mobile alerting and those that did not is reassuring. Second, owing to different levels of granularity between the scores, while we were able to match the high-risk thresholds nearly perfectly on specificity, there was a bigger range for sensitivity at the moderate-risk threshold, particularly for a MEWS score of 3, which only had a sensitivity of 43.7% compared with the range of 50.1% to 52.2% of the others. As a result, the PPV for a MEWS score of 8.2%, which is higher than both EDI and RI, may be misleading. Further, whether these models are identifying deterioration that clinicians are not already aware of is unknown. Finally, while there was considerable diversity among the 7 hospital campuses, they were all geographically located in the Northeast.

## Conclusions

In this cohort study, the performance of AI early warning scores varied widely. eCART outperformed all the scores, and NEWS, which is simple and publicly available, outperformed NEWS2, EDI, RI, and MEWS. Given the wide variation in accuracy, additional transparency and oversight of early warning tools may be warranted.

## Notes

Supplement 1. **eTable 1.** Model Overview **eTable 2.** High-Level Comparison of Model Inputs **eTable 3.** Area Under the Receiver Operator Characteristics (AUROC) Curve for Various Outcomes and Timelines **eTable 4.** Detailed Observation-Level Test Characteristics **eTable 5.** Detailed Encounter-Level Test Characteristics **eFigure.** Receiver Operator Characteristics Curves

Supplement 2.
Data Sharing Statement
