# Error in Author Name

**DOI:** 10.1001/jamanetworkopen.2024.48969

**Published:** 2024-11-07

In the Original Investigation titled “Early Warning Scores With and Without Artificial Intelligence,”^1^ published October 15, 2024, the name of author Zhenqiu Lin, PhD, was misspelled. This article has been corrected.^1^
