# Supplementary material for: Early Warning Scores With and Without Artificial Intelligence
Source: JAMA Netw Open. 2024 Oct 15;7(10):e2438986. doi: 10.1001/jamanetworkopen.2024.38986 (PMC11544488; doi:10.1001/jamanetworkopen.2024.38986)
Supplement: Supplement 1. — eTable 1. Model Overview eTable 2. High-Level Comparison of Model Inputs eTable 3. Area Under the Receiver Operator Characteristics (AUROC) Curve for Various Outcomes and Timelines eTable 4. Detailed Observation-Level Test Characteristics eTable 5. Detailed Encounter-Level Test Characteristics eFigure. Receiver Operator Characteristics Curves [file jamanetwopen-e2438986-s001.pdf]

Supplementary Online Content

Edelson DP. Churpek MM, Carey KA, et al. Early warning scores with and without artificial intelligence. *JAMA Netw Open*. 2024;7(10):e2438986. doi:10.1001/jamanetworkopen.2024.38986

eTable 1. Model Overview

eTable 2. High-Level Comparison of Model Inputs
eTable 3. Area Under the Receiver Operator Characteristics (AUROC) Curve for Various Outcomes and Timelines

eTable 4. Detailed Observation-Level Test Characteristics
eTable 5. Detailed Encounter-Level Test Characteristics
eFigure. Receiver Operator Characteristics Curves

This supplementary material has been provided by the authors to give readers additional information about their work.

© 2024 Edelson DP et al. *JAMA Network Open*.

eTable 1. Model Overview

YNHHS, Yale New Haven Health System; FDA, United States Food and Drug Administration.

Name (Proprietor)	Model specifics	Median Value (Range)	Notes
Modified Early Warning Score (MEWS)[1]	Developed by expert consensus. It requires only five inputs (heart rate, respiratory rate, systolic blood pressure, temperature, and responsiveness) and was intended to be calculated without a computer.	1 (0 - 14)	Most commonly used comparator.[2] Scores are whole numbers.
National Early Warning Score (NEWS) (Royal College of Physicians, London, England)	Consensus modified version of the ViEWS,[3] which was statistically derived using a linear logistic regression model and which is operationalized with whole numbers that can be summed at the bedside, similarly to the MEWS, with the addition of oxygen saturation and supplemental oxygenation. It has established thresholds for moderate (NEWS ≥5) and high-risk (NEWS ≥7).	1 (0 - 20)	NEWS was developed by the Royal College of Physicians and has been endorsed by the National Health Service in the United Kingdom.[4] Scores are whole numbers.
National Early Warning Score 2 (NEWS2) (Royal College of Physicians, London, England)	Consensus modified version of the NEWS which adds a measure of orientation and a separate SpO2 scale for patients with confirmed hypercarbic respiratory failure receiving supplemental oxygen.[5] It uses the same thresholds for moderate	2 (0 - 20)	NEWS2 was developed by the Royal College of Physicians and has been endorsed by the National Health Service in the United Kingdom.

[1] Subbe C, Kruger M, Rutherford P, Gemmel L. Validation of a modified Early Warning Score in medical admissions. *QJM*. 2001;94(10):521-6. doi:10.1093/qjmed/94.10.521

[2] Gerry S, Bonnici T, Birks J, et al. Early warning scores for detecting deterioration in adult hospital patients: systematic review and critical appraisal of methodology. *BMJ*. 2020;369:m1501. doi:10.1136/bmj.m1501

[3] Prytherch D, Smith G, Schmidt P, Featherstone P. ViEWS--Towards a national early warning score for detecting adult inpatient deterioration. Resuscitation. 2010;81(8):932-7. doi:10.1016/j.resuscitation.2010.04.014

[4] Smith G, Prytherch D, Meredith P, Schmidt P, Featherstone P. The ability of the National Early Warning Score (NEWS) to discriminate patients at risk of early cardiac arrest, unanticipated intensive care unit admission, and death. *Resuscitation*. 2013;84(4):465-70. doi:10.1016/j.resuscitation.2012.12.016

[5] Juniper M. NEWS2, patient safety and hypercapnic respiratory failure. *Clinical Medicine*. 2022;22(6):518-521. doi:10.7861/clinmed.2022-0352

© 2024 Edelson DP et al. *JAMA Network Open*.

**Name (Proprietor)**	**Model specifics**	**Median Value (Range)**	**Notes**
	(NEWS2 ≥5) and high-risk (NEWS2 ≥7) as NEWS.		Scores are whole numbers.
Rothman Index (RI) (Spacelabs Healthcare, Snoqualmie, WA)	Heuristic model based on the aggregated 1-year mortality risk of 26 variables.[6]	63 (-58 - 100)	The algorithm received FDA 510(k) marketing clearance (K172959) in 2018. YNHHS turned RI on in production across the health system in 2011. Scores can be whole numbers or decimals, ranging from negative to positive.
Deterioration Index (EDI) (Epic Systems, Verona, WI)	Ordinal logistic regression model based on 17 variables, which was trained to predict mortality within 38 hours or deterioration (defined as ICU transfer, rapid response team (RRT) activation or code blue) within 12 hours.[7]	31.1 (0 - 98.94)	YNHHS turned on EDI in March 2019, scoring every 20 minutes, and ran it alongside RI during the study period with scores and trends visible to clinicians for both scores within the electronic health record. Scores can be integers or decimals.
eCARTv5 (AgileMD, San Francisco, CA)	Gradient-boosted machine learning (GBM) model that uses 97 variables (including 41 vital sign and laboratory value trends).[8] The model was trained in a derivation cohort of 901,491 encounters to predict ward death or direct ward-to-ICU transfer within 8 hours and validated in an independent dataset of 1,975,407 encounters from three health systems, of which Yale New Haven Health System (YNHHS) was one.	44 (0-100)	eCARTv5 received FDA 510(k) clearance (K233253) in June 2024. Scores are integers from 0-99 and can have one or two decimal places between 99 and 100.

[6] Rothman MJ, Rothman SI, Beals J 4th. Development and validation of a continuous measure of patient condition using the Electronic Medical Record. J Biomed Inform. 2013;46(5):837-848. doi:10.1016/j.jbi.2013.06.011

[7] Epic Systems Corporation. Galaxy - Cognitive Computing Model Brief: Deterioration Index. Accessed Aug 21, 2023.

[8] https://www.accessdata.fda.gov/cdrh_docs/pdf23/K233253.pdf

© 2024 Edelson DP et al. *JAMA Network Open*.

Workflow note: For all seven campuses, EDI and RI scores and trend views were visible after scrolling in the Epic Storyboard and could be found in flowsheet documentation, which could then be added to patient lists. In addition, three of the campuses had mobile alerts going to their RRT nurses from the start of the study for "High" and "Very High" RI alerts which were then transitioned to EDI mobile alerts in March 2022 (Campus E) and November 2023 (Campuses A and G), using a threshold of ≥50 or single parameter alerts for oxygen saturation ≤85 or systolic blood pressure ≤ 70. Campus D mobile alerts were also enabled in November 2023 but the other three campuses (B, C and F) never fired mobile alerts during the study period.

© 2024 Edelson DP et al. *JAMA Network Open*.

eTable 2. High-Level Comparison of Model Inputs

Model Variable	Variable Type	MEWS	NEWS	NEWS2	RI	EDI	eCART
Heart Rate	VITALS	✓	✓	✓	✓	✓	✓
Respiratory Rate	VITALS	✓	✓	✓	✓	✓	✓
Systolic BP	VITALS	✓	✓	✓	✓	✓	✓
Temperature	VITALS	✓	✓	✓	✓	✓	✓
Responsiveness/GCS	NURSING ASSESSMENTS	✓	✓	✓	✓	✓	✓
O2 Sat	VITALS		✓	✓	✓	✓	✓
Orientation	NURSING ASSESSMENTS			✓	✓	✓	✓
FiO2/ Supplemental Oxygen	NURSING ASSESSMENTS		✓	✓		✓	✓
BUN	LABORATORY VALUES				✓	✓	✓
Hemoglobin/Hematocrit	LABORATORY VALUES				✓	✓	✓
Potassium	LABORATORY VALUES				✓	✓	✓
Sodium	LABORATORY VALUES				✓	✓	✓
WBC	LABORATORY VALUES				✓	✓	✓
Cardiac	NURSING ASSESSMENTS				✓	✓	
Diastolic BP	VITALS				✓		✓
Chloride	LABORATORY VALUES				✓		✓
Creatinine	LABORATORY VALUES				✓		✓
Braden Score	NURSING ASSESSMENTS				✓		✓
Food/Nutrition	NURSING ASSESSMENTS				✓		
Gastrointestinal	NURSING ASSESSMENTS				✓		
Genitourinary	NURSING ASSESSMENTS				✓		
Musculoskeletal	NURSING ASSESSMENTS				✓		
Peripheral Vascular	NURSING ASSESSMENTS				✓		
Psychosocial	NURSING ASSESSMENTS				✓		
Respiratory	NURSING ASSESSMENTS				✓		
Safety	NURSING ASSESSMENTS				✓		
Skin	NURSING ASSESSMENTS				✓		
pH (ABG & VBG)	LABORATORY VALUES					✓	✓
Platelet Count	LABORATORY VALUES					✓	✓
Age	DEMOGRAPHICS					✓	✓
BMI	NURSING ASSESSMENTS						✓
Urine Output	NURSING ASSESSMENTS						✓
Albumin	LABORATORY VALUES						✓
Alk Phos	LABORATORY VALUES						✓
Anion Gap	LABORATORY VALUES						✓
aPTT	LABORATORY VALUES						✓
Bands	LABORATORY VALUES						✓
Bicarbonate	LABORATORY VALUES						✓
Bilirubin	LABORATORY VALUES						✓
Calcium	LABORATORY VALUES						✓
Eosinophils	LABORATORY VALUES						✓
Glucose	LABORATORY VALUES						✓
INR	LABORATORY VALUES						✓
Lactate	LABORATORY VALUES						✓
Lipase	LABORATORY VALUES						✓

© 2024 Edelson DP et al. *JAMA Network Open*.

Model Variable	Variable Type	MEWS	NEWS	NEWS2	RI	EDI	eCART
Lymphocytes	LABORATORY VALUES						✓
Magnesium	LABORATORY VALUES						✓
MCV	LABORATORY VALUES						✓
Monocytes	LABORATORY VALUES						✓
Neutrophils	LABORATORY VALUES						✓
pCO2 (ABG & VBG)	LABORATORY VALUES						✓
Phosphate	LABORATORY VALUES						✓
pO2 (ABG)	LABORATORY VALUES						✓
RDW	LABORATORY VALUES						✓
SGOT/AST	LABORATORY VALUES						✓
Total Protein	LABORATORY VALUES						✓
ICU During Encounter	ADMINISTRATIVE						✓
Length of Stay	ADMINISTRATIVE						✓
Time of Day	ADMINISTRATIVE						✓

MEWS, Modified Early Warning Score; NEWS, National Early Warning Score; RI, Rothman Index; EDI, Epic Deterioration Index; eCART, eCARTv5.

© 2024 Edelson DP et al. *JAMA Network Open*.

eTable 3. Area Under the Receiver Operator Characteristics (AUROC) Curve for Various Outcomes and Timelines

Outcome	Score	Timeline		
		**12 hour**	**24 hour**	**48 hour**
Deterioration	MEWS	0.772 (0.764, 0.781)	0.757 (0.750, 0.764)	0.741 (0.736, 0.747)
	EDI	0.810 (0.806, 0.818)	0.808 (0.802, 0.812)	0.804 (0.800, 0.808)
	NEWS	0.837 (0.830, 0.843)	0.829 (0.824, 0.835)	0.822 (0.818, 0.826)
	NEWS2	0.837 (0.831, 0.843)	0.831 (0.826, 0.836)	0.826 (0.822, 0.830)
	RI	0.827 (0.821, 0.833)	0.828 (0.823, 0.834)	0.831 (0.827, 0.835)
	eCART	0.906 (0.901, 0.911)	0.895 (0.891, 0.900)	0.885 (0.882, 0.888)
Mortality	MEWS	0.886 (0.873, 0.896)	0.867 (0.859, 0.875)	0.838 (0.833, 0.846)
	EDI	0.913 (0.906, 0.921)	0.905 (0.898, 0.912)	0.891 (0.887, 0.897)
	NEWS	0.932 (0.922, 0.939)	0.921 (0.915, 0.927)	0.902 (0.897, 0.907)
	NEWS2	0.937 (0.930, 0.944)	0.928 (0.923, 0.932)	0.912 (0.908, 0.917)
	RI	0.952 (0.947, 0.957)	0.947 (0.944, 0.951)	0.939 (0.936, 0.941)
	eCART	0.963 (0.958, 0.967)	0.956 (0.952, 0.960)	0.945 (0.942, 0.949)

MEWS, Modified Early Warning Score; NEWS, National Early Warning Score; RI, Rothman Index; EDI, Epic Deterioration Index; eCART, eCARTv5.

© 2024 Edelson DP et al. *JAMA Network Open*.

eTable 4. Detailed Observation-Level Test Characteristics

Score	Threshold	Positivity rate	Sensitivity	Specificity	PPV	NPV
eCART	82	9.4 (9.3, 9.4)	70.3 (69.3, 71.2)	91.3 (91.2, 91.4)	8.3 (8.1, 8.5)	99.6 (99.6, 99.7)
	83	8.8 (8.7, 8.9)	69.3 (68.2, 70.2)	91.9 (91.8, 91.9)	8.7 (8.5, 8.9)	99.6 (99.6, 99.6)
	84	8.3 (8.2, 8.3)	68.2 (67, 69.2)	92.4 (92.4, 92.5)	9.1 (8.9, 9.4)	99.6 (99.6, 99.6)
	85	7.7 (7.6, 7.8)	66.9 (65.7, 67.9)	93 (92.9, 93)	9.6 (9.4, 9.8)	99.6 (99.6, 99.6)
	86	7.2 (7.1, 7.2)	65.7 (64.4, 66.7)	93.5 (93.4, 93.5)	10.2 (9.9, 10.4)	99.6 (99.6, 99.6)
	87	6.6 (6.6, 6.7)	64.3 (63.1, 65.3)	94 (93.9, 94.1)	10.7 (10.4, 10.9)	99.6 (99.6, 99.6)
	88	6.1 (6.1, 6.2)	62.9 (61.8, 64.1)	94.5 (94.4, 94.6)	11.3 (11, 11.6)	99.6 (99.5, 99.6)
	89	5.6 (5.6, 5.7)	61.4 (60.3, 62.7)	95 (94.9, 95)	12.1 (11.7, 12.4)	99.5 (99.5, 99.6)
	90	5.2 (5.1, 5.2)	59.9 (58.8, 61.2)	95.5 (95.4, 95.5)	12.9 (12.5, 13.2)	99.5 (99.5, 99.6)
	91	4.7 (4.6, 4.7)	58.2 (57, 59.4)	95.9 (95.9, 96)	13.7 (13.4, 14.1)	99.5 (99.5, 99.5)
	92	4.2 (4.2, 4.3)	56.4 (55.2, 57.7)	96.4 (96.3, 96.4)	14.8 (14.4, 15.1)	99.5 (99.5, 99.5)
	93	3.8 (3.7, 3.8)	54.4 (53.1, 55.7)	96.8 (96.8, 96.8)	15.9 (15.5, 16.4)	99.5 (99.5, 99.5)
	94	3.3 (3.3, 3.4)	52.2 (51, 53.4)	97.2 (97.2, 97.3)	17.3 (16.9, 17.8)	99.5 (99.4, 99.5)
	95	2.9 (2.8, 2.9)	49.6 (48.4, 50.9)	97.6 (97.6, 97.7)	19 (18.5, 19.5)	99.4 (99.4, 99.5)
	96	2.4 (2.4, 2.5)	46.4 (45, 47.5)	98 (98, 98.1)	20.9 (20.3, 21.5)	99.4 (99.4, 99.4)
	97	2 (2, 2)	42.5 (41.4, 43.9)	98.4 (98.4, 98.5)	23.3 (22.7, 24.2)	99.4 (99.3, 99.4)
	98	1.6 (1.5, 1.6)	37.2 (36.1, 38.6)	98.8 (98.8, 98.9)	26.5 (25.7, 27.4)	99.3 (99.3, 99.3)
	99	1 (1, 1.1)	28.8 (27.9, 30.3)	99.3 (99.3, 99.3)	30.9 (29.7, 32)	99.2 (99.2, 99.2)
EDI	34	27.7 (27.6, 27.8)	73.3 (72.3, 74.2)	72.8 (72.7, 72.9)	2.9 (2.8, 3)	99.6 (99.6, 99.6)
	35	23.8 (23.7, 23.9)	69.9 (68.8, 70.8)	76.7 (76.7, 76.8)	3.2 (3.2, 3.3)	99.6 (99.5, 99.6)
	36	20.3 (20.2, 20.3)	66.5 (65.4, 67.4)	80.2 (80.2, 80.3)	3.6 (3.5, 3.7)	99.5 (99.5, 99.6)
	37	17.2 (17.2, 17.3)	63.2 (62.1, 64.2)	83.3 (83.2, 83.4)	4.1 (3.9, 4.1)	99.5 (99.5, 99.5)
	38	14.6 (14.5, 14.7)	60 (58.7, 61)	85.9 (85.8, 86)	4.5 (4.4, 4.7)	99.5 (99.5, 99.5)
	39	12.4 (12.3, 12.4)	56.6 (55.6, 57.8)	88.1 (88.1, 88.2)	5.1 (4.9, 5.2)	99.5 (99.4, 99.5)
	40	10.5 (10.4, 10.6)	53.6 (52.6, 54.9)	90 (89.9, 90)	5.6 (5.5, 5.8)	99.4 (99.4, 99.4)

© 2024 Edelson DP et al. *JAMA Network Open*.

Score	Threshold	Positivity rate	Sensitivity	Specificity	PPV	NPV
	41	8.9 (8.9, 9)	50.7 (49.4, 51.9)	91.5 (91.5, 91.6)	6.3 (6.1, 6.4)	99.4 (99.4, 99.4)
	42	7.7 (7.6, 7.7)	47.9 (46.5, 49.1)	92.8 (92.7, 92.9)	6.9 (6.7, 7.1)	99.4 (99.4, 99.4)
	43	6.6 (6.5, 6.6)	45.1 (43.8, 46.3)	93.8 (93.8, 93.9)	7.6 (7.3, 7.8)	99.3 (99.3, 99.4)
	44	5.7 (5.7, 5.8)	42.7 (41.3, 43.8)	94.7 (94.6, 94.7)	8.2 (8, 8.5)	99.3 (99.3, 99.3)
	45	5 (4.9, 5)	40.4 (39.1, 41.6)	95.4 (95.4, 95.5)	8.9 (8.6, 9.2)	99.3 (99.3, 99.3)
	46	4.4 (4.3, 4.4)	38 (36.9, 39)	96 (96, 96.1)	9.6 (9.3, 10)	99.3 (99.3, 99.3)
	47	3.9 (3.8, 3.9)	35.9 (34.8, 37)	96.5 (96.5, 96.5)	10.3 (9.9, 10.6)	99.3 (99.2, 99.3)
	48	3.4 (3.4, 3.5)	33.9 (33, 34.9)	96.9 (96.9, 97)	10.9 (10.5, 11.3)	99.2 (99.2, 99.3)
	49	3.1 (3, 3.1)	32 (31.1, 33)	97.3 (97.2, 97.3)	11.6 (11.1, 12)	99.2 (99.2, 99.2)
	50	2.7 (2.7, 2.8)	30.2 (29, 31.2)	97.6 (97.5, 97.6)	12.2 (11.7, 12.6)	99.2 (99.2, 99.2)
	51	2.5 (2.4, 2.5)	28.5 (27.6, 29.6)	97.8 (97.8, 97.8)	12.7 (12.3, 13.2)	99.2 (99.2, 99.2)
	52	2.2 (2.2, 2.3)	26.9 (26.1, 28.1)	98 (98, 98.1)	13.3 (12.9, 13.9)	99.2 (99.2, 99.2)
	53	2 (2, 2.1)	25.4 (24.6, 26.5)	98.2 (98.2, 98.3)	13.9 (13.4, 14.5)	99.2 (99.1, 99.2)
	54	1.8 (1.8, 1.9)	24.2 (23.4, 25.2)	98.4 (98.4, 98.4)	14.5 (14, 15.2)	99.1 (99.1, 99.2)
	55	1.7 (1.7, 1.7)	22.8 (21.9, 23.8)	98.6 (98.5, 98.6)	15 (14.5, 15.7)	99.1 (99.1, 99.2)
	56	1.5 (1.5, 1.6)	21.7 (20.7, 22.7)	98.7 (98.7, 98.7)	15.6 (15, 16.4)	99.1 (99.1, 99.1)
	57	1.4 (1.4, 1.4)	20.6 (19.7, 21.7)	98.8 (98.8, 98.8)	16.2 (15.6, 17.1)	99.1 (99.1, 99.1)
	58	1.3 (1.3, 1.3)	19.5 (18.6, 20.5)	98.9 (98.9, 98.9)	16.9 (16.1, 17.7)	99.1 (99.1, 99.1)
	59	1.2 (1.1, 1.2)	18.6 (17.7, 19.4)	99 (99, 99)	17.5 (16.7, 18.4)	99.1 (99.1, 99.1)
MEWS	2	20.9 (20.8, 21)	65.7 (64.4, 67)	79.6 (79.5, 79.7)	3.5 (3.4, 3.6)	99.5 (99.5, 99.5)
	3	5.9 (5.8, 5.9)	43.7 (42.5, 44.9)	94.6 (94.5, 94.6)	8.2 (8, 8.5)	99.3 (99.3, 99.4)
	4	1.8 (1.8, 1.8)	27.6 (26.4, 28.6)	98.5 (98.5, 98.5)	16.9 (16.2, 17.5)	99.2 (99.2, 99.2)
	5	0.7 (0.7, 0.7)	16.3 (15.6, 17.3)	99.5 (99.5, 99.5)	26 (24.8, 27.3)	99.1 (99, 99.1)
NEWS	3	20.5 (20.4, 20.5)	73.5 (72.3, 74.4)	80.1 (80, 80.2)	4 (3.9, 4)	99.6 (99.6, 99.6)
	4	10.9 (10.8, 10.9)	62 (61, 63.1)	89.7 (89.6, 89.8)	6.3 (6.2, 6.5)	99.5 (99.5, 99.6)
	5	6 (5.9, 6)	51.5 (50.4, 52.8)	94.5 (94.5, 94.6)	9.5 (9.3, 9.8)	99.4 (99.4, 99.5)

© 2024 Edelson DP et al. *JAMA Network Open*.

Score	Threshold	Positivity rate	Sensitivity	Specificity	PPV	NPV
	6	3.2 (3.2, 3.3)	40.7 (39.6, 42.4)	97.2 (97.2, 97.2)	14 (13.5, 14.4)	99.3 (99.3, 99.4)
	7	1.8 (1.8, 1.8)	31.5 (30.7, 32.9)	98.5 (98.5, 98.5)	19.1 (18.5, 20)	99.2 (99.2, 99.3)
	8	1.1 (1.1, 1.1)	23.8 (22.9, 25.3)	99.2 (99.2, 99.2)	24.5 (23.5, 25.6)	99.1 (99.1, 99.2)
NEWS2	4	18.8 (18.7, 18.9)	70.8 (69.9, 71.9)	81.8 (81.7, 81.9)	4.2 (4.1, 4.3)	99.6 (99.6, 99.6)
	5	11.3 (11.2, 11.4)	61.1 (60, 62.2)	89.2 (89.2, 89.3)	6 (5.8, 6.1)	99.5 (99.5, 99.5)
	6	6.3 (6.3, 6.4)	50.1 (49, 51.4)	94.2 (94.1, 94.2)	8.7 (8.5, 9)	99.4 (99.4, 99.4)
	7	3.6 (3.6, 3.7)	39.9 (38.8, 41.1)	96.8 (96.7, 96.8)	12.1 (11.7, 12.6)	99.3 (99.3, 99.3)
	8	2.1 (2.1, 2.2)	30.5 (29.6, 32.1)	98.2 (98.1, 98.2)	15.8 (15.1, 16.4)	99.2 (99.2, 99.2)
	9	1.2 (1.2, 1.2)	22.4 (21.4, 23.5)	99.1 (99, 99.1)	21 (19.9, 21.9)	99.1 (99.1, 99.2)
RI	20	1.2 (1.2, 1.2)	22.5 (21.8, 23.4)	99 (99, 99)	20.2 (19.5, 21.2)	99.1 (99.1, 99.2)
	21	1.4 (1.3, 1.4)	23.9 (23.1, 24.8)	98.9 (98.9, 98.9)	19.3 (18.7, 20.2)	99.1 (99.1, 99.2)
	22	1.5 (1.5, 1.5)	25.2 (24.4, 26.3)	98.8 (98.7, 98.8)	18.5 (17.8, 19.2)	99.2 (99.1, 99.2)
	23	1.7 (1.7, 1.7)	26.6 (25.7, 27.6)	98.6 (98.6, 98.6)	17.5 (16.8, 18.1)	99.2 (99.2, 99.2)
	24	1.9 (1.8, 1.9)	27.9 (27, 29)	98.4 (98.4, 98.5)	16.6 (15.9, 17.1)	99.2 (99.2, 99.2)
	25	2.1 (2, 2.1)	29.4 (28.4, 30.4)	98.2 (98.2, 98.3)	15.7 (15.2, 16.3)	99.2 (99.2, 99.2)
	26	2.3 (2.2, 2.3)	30.8 (29.9, 31.9)	98 (98, 98.1)	14.9 (14.4, 15.5)	99.2 (99.2, 99.2)
	27	2.5 (2.5, 2.6)	32.3 (31.4, 33.3)	97.8 (97.8, 97.8)	14.1 (13.6, 14.6)	99.2 (99.2, 99.3)
	28	2.8 (2.8, 2.8)	33.9 (32.9, 34.9)	97.6 (97.5, 97.6)	13.4 (12.9, 13.9)	99.2 (99.2, 99.3)
	29	3.1 (3, 3.1)	35.3 (34.3, 36.4)	97.3 (97.3, 97.3)	12.7 (12.3, 13.1)	99.3 (99.2, 99.3)
	30	3.4 (3.3, 3.4)	36.8 (35.8, 37.9)	97 (97, 97)	12 (11.6, 12.4)	99.3 (99.3, 99.3)
	31	3.7 (3.7, 3.7)	38.2 (37.3, 39.3)	96.7 (96.6, 96.7)	11.4 (11, 11.7)	99.3 (99.3, 99.3)
	32	4.1 (4, 4.1)	39.6 (38.6, 40.7)	96.3 (96.3, 96.4)	10.8 (10.4, 11.1)	99.3 (99.3, 99.3)
	33	4.4 (4.4, 4.5)	40.9 (39.9, 41.9)	96 (95.9, 96)	10.2 (9.9, 10.5)	99.3 (99.3, 99.3)
	34	4.8 (4.8, 4.9)	42.1 (41.2, 43.2)	95.6 (95.6, 95.6)	9.6 (9.3, 9.9)	99.3 (99.3, 99.4)
	35	5.2 (5.2, 5.3)	43.5 (42.6, 44.6)	95.2 (95.2, 95.2)	9.1 (8.9, 9.4)	99.3 (99.3, 99.4)
	36	5.7 (5.6, 5.7)	44.8 (43.6, 46)	94.7 (94.7, 94.8)	8.7 (8.4, 8.9)	99.4 (99.3, 99.4)

© 2024 Edelson DP et al. *JAMA Network Open*.

Score	Threshold	Positivity rate	Sensitivity	Specificity	PPV	NPV
	37	6.1 (6.1, 6.2)	46.1 (44.9, 47.3)	94.3 (94.3, 94.3)	8.3 (8, 8.5)	99.4 (99.3, 99.4)
	38	6.6 (6.6, 6.7)	47.3 (46.3, 48.5)	93.8 (93.8, 93.9)	7.9 (7.6, 8.1)	99.4 (99.4, 99.4)
	39	7.1 (7.1, 7.2)	48.8 (47.7, 50)	93.3 (93.3, 93.4)	7.5 (7.3, 7.7)	99.4 (99.4, 99.4)
	40	7.7 (7.6, 7.7)	50.1 (49, 51.1)	92.8 (92.7, 92.8)	7.2 (7, 7.4)	99.4 (99.4, 99.4)
	41	8.2 (8.2, 8.3)	51.2 (50.2, 52.2)	92.3 (92.2, 92.3)	6.9 (6.7, 7)	99.4 (99.4, 99.4)
	42	8.8 (8.7, 8.9)	52.3 (51.4, 53.4)	91.7 (91.6, 91.7)	6.6 (6.4, 6.7)	99.4 (99.4, 99.4)
	43	9.4 (9.4, 9.5)	53.7 (52.6, 54.7)	91.1 (91, 91.1)	6.3 (6.1, 6.5)	99.4 (99.4, 99.5)
	44	10 (10, 10.1)	55 (54, 56)	90.5 (90.4, 90.5)	6 (5.9, 6.2)	99.4 (99.4, 99.5)
	45	10.7 (10.7, 10.8)	56.3 (55.2, 57.3)	89.8 (89.7, 89.9)	5.8 (5.6, 5.9)	99.5 (99.4, 99.5)
	46	11.4 (11.3, 11.5)	57.5 (56.5, 58.4)	89.1 (89.1, 89.2)	5.6 (5.4, 5.7)	99.5 (99.5, 99.5)
	47	12.1 (12.1, 12.2)	58.7 (57.7, 59.6)	88.4 (88.3, 88.5)	5.3 (5.2, 5.5)	99.5 (99.5, 99.5)
	48	12.9 (12.8, 12.9)	59.8 (58.7, 60.8)	87.6 (87.6, 87.7)	5.1 (5, 5.3)	99.5 (99.5, 99.5)
	49	13.7 (13.6, 13.7)	61.1 (60, 62.1)	86.9 (86.8, 86.9)	4.9 (4.8, 5.1)	99.5 (99.5, 99.5)
	50	14.5 (14.4, 14.5)	62.4 (61.2, 63.5)	86 (86, 86.1)	4.7 (4.6, 4.9)	99.5 (99.5, 99.5)
	51	15.4 (15.3, 15.4)	63.5 (62.5, 64.5)	85.2 (85.1, 85.2)	4.6 (4.5, 4.7)	99.5 (99.5, 99.5)
	52	16.3 (16.2, 16.3)	64.6 (63.5, 65.6)	84.3 (84.2, 84.3)	4.4 (4.3, 4.5)	99.5 (99.5, 99.5)
	53	17.2 (17.2, 17.3)	65.9 (64.7, 66.8)	83.3 (83.3, 83.4)	4.2 (4.1, 4.3)	99.5 (99.5, 99.6)
	54	18.2 (18.2, 18.3)	67.1 (65.9, 68)	82.3 (82.3, 82.4)	4.1 (4, 4.2)	99.6 (99.5, 99.6)
	55	19.3 (19.2, 19.3)	68.3 (67.1, 69.3)	81.3 (81.2, 81.3)	3.9 (3.8, 4)	99.6 (99.5, 99.6)
	56	20.4 (20.3, 20.4)	69.4 (68.4, 70.5)	80.2 (80.1, 80.2)	3.8 (3.7, 3.9)	99.6 (99.6, 99.6)
	57	21.5 (21.5, 21.6)	70.7 (69.6, 71.5)	79 (78.9, 79.1)	3.6 (3.5, 3.7)	99.6 (99.6, 99.6)

MEWS, Modified Early Warning Score; NEWS, National Early Warning Score; RI, Rothman Index; EDI, Epic Deterioration Index; eCART, eCARTv5.

© 2024 Edelson DP et al. *JAMA Network Open*.

eTable 5. Detailed Encounter-Level Test Characteristics

Score	Threshold	Encounters with Alerts	Deteriorations Identified	Time to deterioration
eCART	82	156814 (43.2%)	15671 (93.9%)	46 (11, 137)
	83	151012 (41.6%)	15613 (93.5%)	45 (10, 135)
	84	144965 (39.9%)	15558 (93.2%)	44 (10, 134)
	85	138660 (38.2%)	15469 (92.7%)	43 (9, 131)
	86	132228 (36.4%)	15373 (92.1%)	41 (9, 129)
	87	125757 (34.7%)	15265 (91.4%)	40 (8, 126)
	88	119016 (32.8%)	15169 (90.9%)	38 (7, 123)
	89	112164 (30.9%)	15043 (90.1%)	36 (6, 120)
	90	104890 (28.9%)	14906 (89.3%)	33 (5, 115)
	91	97630 (26.9%)	14713 (88.1%)	31 (4, 111)
	92	89992 (24.8%)	14520 (87%)	29 (4, 107)
	93	82059 (22.6%)	14272 (85.5%)	26 (3, 101)
	94	73761 (20.3%)	13989 (83.8%)	22 (2, 94)
	95	65232 (18%)	13674 (81.9%)	19 (1, 88)
	96	56373 (15.5%)	13269 (79.5%)	15 (1, 79)
	97	46682 (12.9%)	12711 (76.1%)	11 (0, 69)
	98	36378 (10%)	11879 (71.2%)	6 (0, 53)
	99	24606 (6.8%)	10239 (61.3%)	2 (0, 30)
EDI	34	211685 (58.3%)	15499 (92.8%)	51 (13, 146)
	35	198172 (54.6%)	15315 (91.7%)	49 (11, 142)
	36	184474 (50.8%)	15082 (90.3%)	46 (10, 139)
	37	171036 (47.1%)	14835 (88.9%)	43 (8, 135)
	38	157789 (43.5%)	14578 (87.3%)	40 (7, 131)
	39	145162 (40%)	14287 (85.6%)	38 (5, 126)
	40	132957 (36.6%)	14005 (83.9%)	34 (4, 121)
	41	121471 (33.5%)	13685 (82%)	31 (3, 116)
	42	110947 (30.6%)	13368 (80.1%)	27 (2, 110)
	43	101441 (28%)	13060 (78.2%)	24 (1, 104)
	44	92518 (25.5%)	12701 (76.1%)	21 (0, 97)
	45	84298 (23.2%)	12359 (74%)	18 (0, 91)
	46	76948 (21.2%)	11975 (71.7%)	15 (0, 86)
	47	70265 (19.4%)	11591 (69.4%)	12 (0, 80)
	48	64294 (17.7%)	11217 (67.2%)	9 (0, 74)
	49	58809 (16.2%)	10840 (64.9%)	7 (0, 68)
	50	53879 (14.8%)	10470 (62.7%)	5 (0, 62)
	51	49347 (13.6%)	10082 (60.4%)	3 (0, 56)
	52	45250 (12.5%)	9693 (58.1%)	2 (0, 50)
	53	41538 (11.4%)	9341 (56%)	1 (0, 44)

© 2024 Edelson DP et al. *JAMA Network Open*.

Score	Threshold	Encounters with Alerts	Deteriorations Identified	Time to deterioration
	54	37996 (10.5%)	8967 (53.7%)	1 (0, 39)
	55	34922 (9.6%)	8594 (51.5%)	0 (0, 34)
	56	32078 (8.8%)	8204 (49.1%)	0 (0, 29)
	57	29344 (8.1%)	7824 (46.9%)	0 (0, 25)
	58	26910 (7.4%)	7475 (44.8%)	0 (0, 21)
	59	24626 (6.8%)	7112 (42.6%)	0 (0, 17)
MEWS	2	259356 (71.5%)	15668 (93.9%)	50 (13, 143)
	3	131572 (36.3%)	13792 (82.6%)	26 (2, 106)
	4	60547 (16.7%)	10828 (64.9%)	5 (0, 56)
	5	27093 (7.5%)	7572 (45.4%)	0 (0, 15)
NEWS	3	227171 (62.6%)	15707 (94.1%)	51 (13, 144)
	4	162504 (44.8%)	14960 (89.6%)	42 (8, 129)
	5	115301 (31.8%)	14055 (84.2%)	31 (4, 110)
	6	78088 (21.5%)	12785 (76.6%)	18 (0, 87)
	7	51491 (14.2%)	11238 (67.3%)	8 (0, 63)
	8	33557 (9.2%)	9542 (57.2%)	2 (0, 39)
NEWS2	4	194371 (53.6%)	15457 (92.6%)	48 (12, 140)
	5	148367 (40.9%)	14762 (88.4%)	40 (7, 128)
	6	107208 (29.5%)	13719 (82.2%)	28 (3, 108)
	7	74035 (20.4%)	12443 (74.5%)	17 (0, 86)
	8	50512 (13.9%)	10924 (65.4%)	6 (0, 62)
	9	32891 (9.1%)	9071 (54.3%)	1 (0, 37)
RI	57	149287 (41.1%)	14529 (87%)	45 (8, 139)
	56	143770 (39.6%)	14400 (86.3%)	44 (7, 138)
	55	138266 (38.1%)	14250 (85.4%)	42 (6, 135)
	54	133093 (36.7%)	14095 (84.4%)	41 (6, 134)
	53	128120 (35.3%)	13945 (83.5%)	39 (5, 132)
	52	123157 (33.9%)	13770 (82.5%)	38 (4, 130)
	51	118334 (32.6%)	13616 (81.6%)	37 (4, 129)
	50	113595 (31.3%)	13465 (80.7%)	35 (3, 127)
	49	109036 (30%)	13283 (79.6%)	33 (2, 124)
	48	104476 (28.8%)	13096 (78.5%)	32 (2, 122)
	47	100006 (27.6%)	12912 (77.3%)	30 (1, 119)
	46	95741 (26.4%)	12734 (76.3%)	28 (1, 118)
	45	91679 (25.3%)	12567 (75.3%)	26 (0, 115)
	44	87519 (24.1%)	12395 (74.3%)	25 (0, 111)
	43	83737 (23.1%)	12194 (73%)	23 (0, 109)
	42	79886 (22%)	11989 (71.8%)	22 (0, 107)
	41	76287 (21%)	11805 (70.7%)	20 (0, 104)
	40	72739 (20%)	11603 (69.5%)	19 (0, 101)

© 2024 Edelson DP et al. *JAMA Network Open*.

Score	Threshold	Encounters with Alerts	Deteriorations Identified	Time to deterioration
	39	69298 (19.1%)	11409 (68.3%)	17 (0, 99)
	38	66056 (18.2%)	11207 (67.1%)	15 (0, 95)
	37	62787 (17.3%)	10975 (65.7%)	13 (0, 92)
	36	59673 (16.4%)	10760 (64.5%)	12 (0, 88)
	35	56572 (15.6%)	10536 (63.1%)	10 (0, 85)
	34	53633 (14.8%)	10302 (61.7%)	9 (0, 82)
	33	50819 (14%)	10075 (60.4%)	7 (0, 78)
	32	48032 (13.2%)	9863 (59.1%)	6 (0, 74)
	31	45375 (12.5%)	9641 (57.8%)	4 (0, 71)
	30	42761 (11.8%)	9414 (56.4%)	3 (0, 67)
	29	40321 (11.1%)	9158 (54.9%)	2 (0, 62)
	28	37922 (10.4%)	8931 (53.5%)	2 (0, 58)
	27	35621 (9.8%)	8718 (52.2%)	1 (0, 54)
	26	33368 (9.2%)	8485 (50.8%)	0 (0, 50)
	25	31148 (8.6%)	8187 (49%)	0 (0, 46)
	24	29036 (8%)	7927 (47.5%)	0 (0, 42)
	23	27076 (7.5%)	7692 (46.1%)	0 (0, 38)
	22	25190 (6.9%)	7449 (44.6%)	0 (0, 33)
	21	23446 (6.5%)	7199 (43.1%)	0 (0, 29)
	20	21697 (6%)	6941 (41.6%)	0 (0, 26)

MEWS, Modified Early Warning Score; NEWS, National Early Warning Score; RI, Rothman Index; EDI, Epic Deterioration Index; eCART, eCARTv5.

© 2024 Edelson DP et al. *JAMA Network Open*.

eFigure. Receiver Operator Characteristics Curves

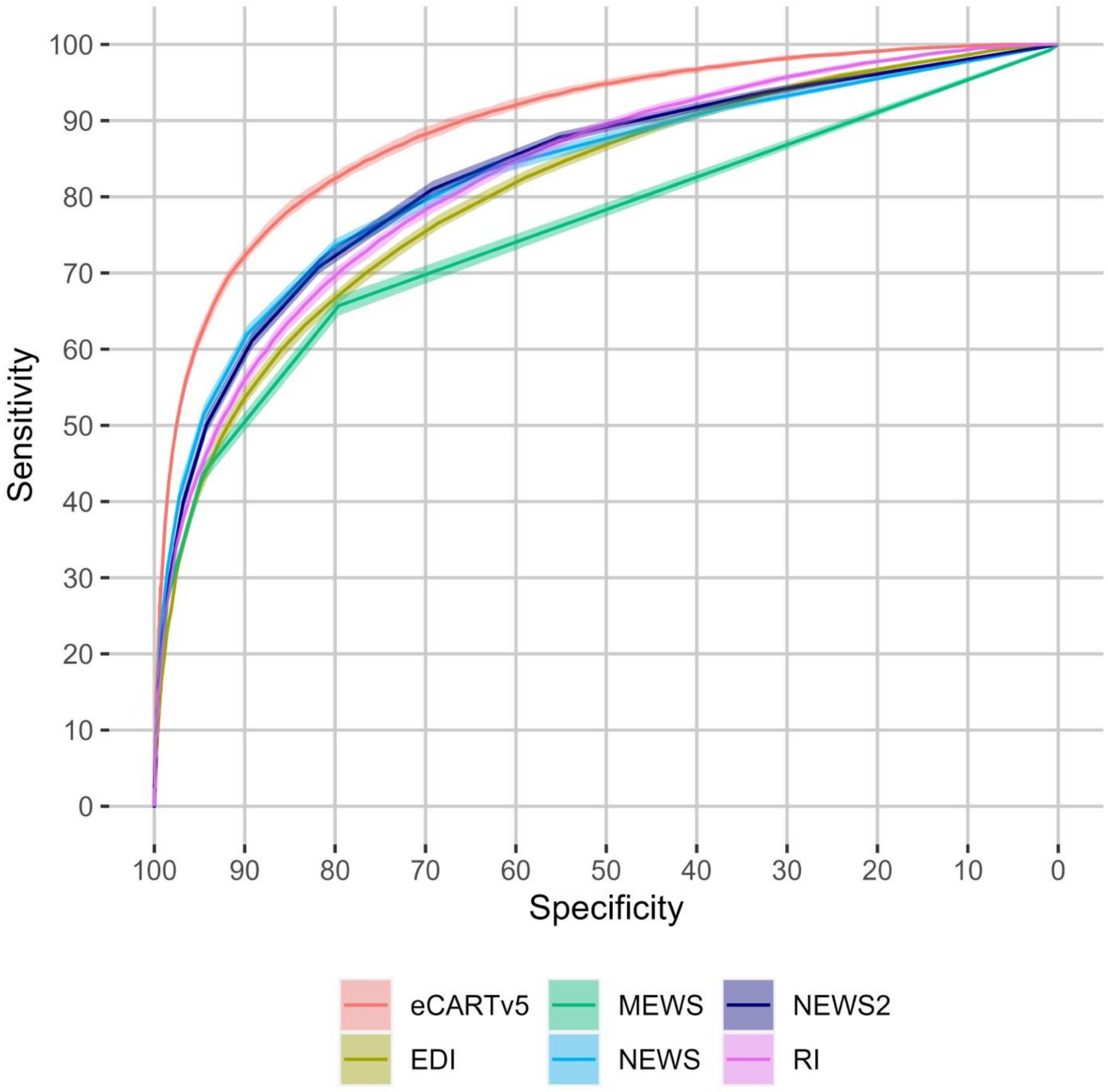

MEWS, Modified Early Warning Score; NEWS, National Early Warning Score; RI, Rothman Index; EDI, Epic Deterioration Index; eCART, eCARTv5. Shading represents the 95% confidence intervals.

© 2024 Edelson DP et al. *JAMA Network Open*.
