# Supplementary material for: Early Warning Scores With and Without Artificial Intelligence
Source: JAMA Netw Open. 2024 Oct 15;7(10):e2438986. doi: 10.1001/jamanetworkopen.2024.38986 (PMC11544488; doi:10.1001/jamanetworkopen.2024.38986)
Supplement: Supplement 2. — Data Sharing Statement [file jamanetwopen-e2438986-s002.pdf]

Data Sharing Statement

Edelson. Early Warning Scores With and Without Artificial Intelligence. *JAMA Netw Open*. Published October 15, 2024. doi:10.1001/jamanetworkopen.2024.38986

Data
Data available: No
